# Skeletal determinants of tail length are different between macaque species groups

**DOI:** 10.1038/s41598-018-37963-z

**Published:** 2019-02-04

**Authors:** Hikaru Wakamori, Yuzuru Hamada

**Affiliations:** Primate Research Institute, Department of Biology, Faculty of Science, Kyoto University, Inuyama, Aichi, 484-8506 Japan

## Abstract

Macaques (genus *Macaca*) are known to have wide variation in tail length. Within each species group tail length varies, which could be associated with a phylogenetic trend seen in caudal vertebral morphology. We compared numbers and lengths of caudal vertebrae in species of the *fascicularis* group, *M*. *assamensis* (*sinica* group), *M*. *nemestrina* (*silenus* group), and those obtained from reports for an additional 11 species. Our results suggest different trends in number and lengths. The caudal vertebral length profiles revealed upward convex patterns for macaques with relative tail lengths of ≥15%, and flat to decreasing for those with relative tail lengths of ≤12%. They varied between species groups in terms of the lengths of proximal vertebrae, position and length of the longest vertebra, numbers and lengths of distal vertebrae, and total number of vertebrae. In *silenus* and *sinica* group, the vertebral length is the major skeletal determinant of tail length. On the other hand, the vertebral number is the skeletal determinant of tail length in the *fascicularis* group. Tail length variation among species groups are caused by different mechanisms which reflect the evolutionary history of macaques.

## Introduction

The macaques (genus *Macaca*) are known for its great variation of tail length^1–7^. Tail length is commonly evaluated by a relative measure (relative tail length: RTL in %) which is tail length standardized by the head-body length (HBL) to cancel the effect of body size^7–9^. The macaques comprise of four species groups: *fascicularis*, *sinica*, *silenus*, and *sylvanus* groups^10^, and there are as many as 23 species (including *Macaca leucogenys*)^11^. RTL ranges from 2% to 124% among all macaque species with overlap within the four species groups^1–7^. From an evolutionary perspective, these tail length variations have been considered as adaptations due to thermoregulation and/or with necessity as a balancing mechanism during locomotor activity among others^12,13^. Macaques are widely distributed in Asia, except for *M*. *sylvanus* that is distributed in north-west Africa^14^, and show latitudinal cline in tail length^1,15^ that is species living in the south tend to have longer tails compared to species living in the north. As a locomotor balancer, arboreal pronograde quadrupeds tend to have longer tails than terrestrial counterparts^13,16^. Macaques are semi-terrestrial in general^17^ with various inclinations to either arboreality or terrestriality. However, what kinds of mechanisms work to change the tail length?

The tail is composed of caudal vertebrae that are linearly arranged, and the number and length of caudal vertebrae are the skeletal determinants of tail length. Primates’ caudal vertebrae are categorized into three types based on their morphology: proximal, transition, and distal^8,18–20^. Among these three morphology types the muscular and tendon attachments are different^21,22^. The proximal vertebrae are the main vertebrae that enables large joint motion at the base of tail and contribute on tail swinging, thus the region have been studied for the estimation of tail length^16,20^. Tojima^20^ suggested that the number of proximal vertebrae represents tail length; and Russo^16^ showed that the length of proximal vertebrae, especially the transition vertebra, is a good estimator of the tail length. However, there is no evidence that tail length is determined only by proximal vertebrae, and Hamada *et al*.^19^ reported that total caudal vertebrae number more correlates with the number of caudal vertebrae in distal region than proximal region. Though distal vertebrae do not swing wide between adjacent vertebrae, their length decreases distally from the longest vertebra and they could function as elements of cantilever, which is important in balancing. The number of vertebrae and vertebral lengths independently change in some mammals; for example, Rutledge *et al*.^23^ reported on two replicate lines of mice with increased tail length, one of which had an increased number of vertebrae and the other had an increased vertebral length. Another study reported by Kingsley *et al*.^24^ on the North American deer mouse, which exhibits wide tail length variations, found two tail elongation mechanisms; increase of the number or elongation of vertebrae, which were controlled by separate genetic loci^24^. Thus both skeletal determinants, number and length of caudal vertebrae, need to be investigated at the same time.

Though the sample sizes were limited, caudal vertebrae measurements were done by Fooden in each species group, separately^1–6,25^. In Fooden’s last study, he suggested that homologous genetic factors may control the tail length reduction in *fascicularis*, *sinica* and *silenus* groups^1^. However, there have been no reports comparing across the species groups. Since macaques in Asia diverged into species groups approximately 3.8 million years ago^26–28^, the intra-species group tail length variation should have occurred after the species group divergence. From the recent study of Sehner *et al*., they have reported that primates’ relative tail length have been changed both in increase and decrease^29^. Most of the changes occurred in Old World primates and the genus *Macaca* was the notably high in changes, those were four increases and three decreases^29^. They mentioned that the tail increase were seen in three clades separately; (a) *M*. *nemestrina*, *M*. *silenus*, *M*. *pagensis*, and *M*. *leonine*; (b) *M*. *sinica*, *M*. *radiata*, and *M*. *munzala*; (c) *M*. *cyclopis*, *M*. *mulatta*, and *M*. *fascicularis*^29^, which exactly follows the species group; (a) *silenus* group, (b) *sinica* group, and (c) *fascicularis* group. From these studies we hypothesize there could be species groups’ trend in number and length of caudal vertebrae. Since the function differs in caudal vertebrae, such as proximal vertebrae are acting as the foundation for movement and distal vertebrae are responsible for balancing mechanism by its weight, the comparison of number and length in different vertebrae (proximal, transition, and distal) are essential information to consider macaques’ tail evolution and adaptation. The caudal vertebral number and length could have phylogenetic trends and/or characteristics dependent on tail length.

In this study we compared the number and length of caudal vertebrae of 16 taxa of macaques. We focused on whether species group trends are apparent or not, especially by comparing similar tail length species in different species groups. Tail length variation occurred more by vertebral length changes in *silenus* and *sinica* groups, whereas the *fascicularis* group showed a greater change in vertebral number. We discuss the relation of caudal vertebrae and tail function for balancing mechanism, the similarity in the long-tailed species, and how tail length variation occurred in each species group by taking molecular phylogeny into consideration.

## Materials and Methods

We obtained measurements of the caudal vertebrae from skeletal specimens stored at the Kyoto University Primate Research Institute (KUPRI) and the Japan Monkey Centre (JMC), selecting only adults based on their complete epiphyseal fusion. We measured the vertebral lengths in six species of macaques: Japanese macaque (*M*. *fuscata*), rhesus macaque (*M*. *mulatta*), Taiwanese macaque (*M*. *cyclopis*), and cynomolgus monkeys (*M*. *fascicularis*) of the *fascicularis* group; Assamese macaque (*M*. *assamensis assamensis*) of the *sinica* group; and southern pig-tail macaque (*M*. *nemestrina*) of the *silenus* group (Table 1). The *M*. *fuscata* samples included only the nominotypical subspecies *M*. *f*. *fuscata*. We treated the eastern (China and its vicinity) and western (India and its vicinity) groups of *M*. *mulatta* separately because of the differences in tail length^2,30^.

**Table 1:** The samples used in this study.

Species group	*Macaca* spp.	Specimen sample number			Reference sample number			Total
		♀	♂	Total	♀	♂	Total	
*fascicularis*	*M*. *fuscata*^#1^	9	7	16				16
	*M*. *mulatta* (East)	4	3	7				7
	(West)	5	5	10				10
	*M*. *cyclopis*	9	10	19				19
	*M*. *fascicularis*	8	8	16				16
*sinica*	*M*. *arctoides*				4	6	10^*1^	10
	*M*. *thibetana*				1	2	3^*2^	3
	*M*. *assamensis*^#2^	4	5	9		2	2^*2^	11
	*M*. *radiata*					1	1^*2^	1
	*M*. *sinica*					2	2^*2^	2
*silenus*	*M*. *nigra*				3	2	5^*3^	5
	*M*. *maura*				3	4	7^*3^	7
	*M*. *ochreata*^#3^					1	1^*3^	1
	*M*. *leonina*				2	3	5^*4^	5
	*M*. *nemestrina*	10	6	16		8	8^*4^	24
	*M*. *silenus*				3	1	4^*4^	4
Total	15 species	49	44	**93**	16	32	48	**141**

The subspecies are as follows; ^#1^*M. f. fuscata*; ^#2^*M. a. assamensis*; ^#3^*M. ochreata brunnescens*.

The references were cited from the literature as follows.

*^1^Fooden, 1990; *^2^Fooden, 1988; *^3^Fooden, 1969; *^4^Fooden 1975.

For each taxon (or group for the rhesus), we counted the number of vertebrae in each region and calculated the median (Table 2). We measured the cranio-caudal length of vertebral body to the nearest 0.1 mm using a digital sliding caliper (Mitutoyo Corporation). Each vertebra was measured three times for accuracy, and the median value was taken for analysis. We included only individuals with three sacral vertebrae, as three is the common count of the sacral vertebrae in macaques^31–33^. Lumbar sacralization was not observed in specimens used. To expand taxonomic breadth, we utilized vertebral measurements from Fooden^3–6^ (Table 1).

**Table 2:** Median number of caudal vertebrae, with the range in parentheses, for each region and the total number; mean relative tail length (RTL); mean single caudal vertebral length, average longest caudal vertebral length standardized by head and body length (LV length/HBL), and aspect ratio.

Species group	*Macaca* spp.	Regions				Total number of caudal vertebrae	RTL (%)	Mean single caudal vertebral length (%)^*9^	LV length/HBL (%)	Aspect ratio (LV length/Total number of caudal vertebrae)
		proximal	transitional	proximal- transitional^*7^	distal					
*fascicularis*	*M*. *fuscata*^#1^	4 (3–4)	1.5 (1–3)	5 (5–6)	6 (5–7)	11 (10–12)	15^*5^	1.82	2.71	0.247
	*M*. *mulatta* (East)	4 (4–5)	2 (1–2)	6 (5–6)	10 (8–12)	16 (14–17)	35^*6^	2.56	4.45	0.278
	(West)	4 (4–5)	2 (1–2)	6 (5–7)	10 (10–12)	17 (15–18)	45^*6^	2.71	4.54	0.267
	*M*. *cyclopis*	5 (4–5)	2 (1–3)	7 (6–8)	14 (13–16)	21 (20–23)	84^*5^	3.74	6.31	0.301
	*M*. *fascicularis*	5 (4–6)	2 (1–3)	7.5 (7–8)	18 (16–20)	26 (24–27)	117^*5^	4.30	7.22	0.278
*sinica*	*M*. *arctoides*			3 (1–5)^*1^	6.5 (3–10)^*1^	9 (6–11)^*1^	8^*1^	1.36	1.86	0.206
	*M*. *thibetana*			5 (1–6)^*2^	8 (4–11)^*2^	12 (10–13)^*2^	12^*2^	1.47	1.98	0.165
	*M*. *assamensis*^#2^	5 (4–5)	2 (1–3)	7 (6–8)^*2^	♀8.5 (6–10)^*2,8^ ♂11 (11–13)^*2,8^	18 (13–19)^*2,8^	38^*2^	2.50	4.11	0.228
	*M*. *radiata*			7^*2^	19^*2^	26^*2^	108^*2^	4.28	7.50	0.288
	*M*. *sinica*			8^*2^	17^*2^	25^*2^	124^*2^	4.58	7.66	0.306
*silenus*	*M*. *nigra*					6 (5–8)^*3^	4^*3^	0.97	1.40	0.233
	*M*. *maura*					8 (8–11)^*3^	7^*3^	1.06	1.55	0.194
	*M*. *ochreata*^#3^					9^*3^	9^*3^	1.18	1.85	0.205
	*M*. *leonina*			7 (7–8)^*4^	10 (10–12)^*4^	17 (17–20)^*4^	37^*4^	2.25	3.77	0.222
	*M*. *nemestrina*	5 (4–5)	2 (2–3)	7 (6–9)^*4^	11 (8–15)^*4^	18 (14–22)^*4^	37^*4^	1.84	3.05	0.170
	*M*. *silenus*			7.5 (7–8)^*4^	13.5 (13–14)^*4^	21^*4^	66^*4^	2.78	4.56	0.217

The subspecies are as follows; ^#1^*M. f. fuscata*; ^#2^*M. a. assamensis*; ^#3^*M. ochreata brunnescens*.

The references and mean RTL were cited and calculated from the literature as follows.

*^1^Fooden, 1990; *^2^Fooden, 1988; *^3^Fooden, 1969; *^4^Fooden 1975; *^5^Fooden, 2006, *^6^Fooden, 2000.

*^7^The number of proximal-transitional regions equal to the number of proximal plus transitional regions, and equal to the ordinal number of the longest vertebra.

*^8^There were statistical differences between the sexes. The result of Welch Two Sample *t*-test by species sex.

*M*. *a*. *assamensis*: distal (number of caudal vertebrae in distal region) *t* = −3.553, df = 4.306, *P*-value = 0.021; Total Number of caudal vertebrae *t* = −3.164, df = 4.148, *P*-value = 0.03237.

*^9^Mean single caudal vertebral length were calculated as each specie’s mean total length/HBL/total number of caudal vertebrae, which is the value in Supplementary Table S3.

**Figure 1:**
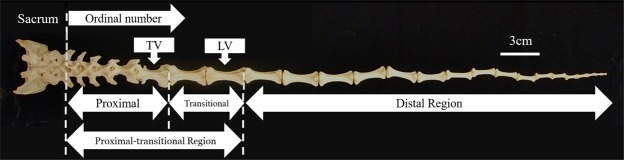
Articulated sacrum and caudal vertebrae. TV: transition vertebra; LV: longest vertebra. The caudal vertebrae can be divided into three regions (proximal, transitional and distal). The proximal-transitional region is the region from the first caudal vertebrae up to the LV.

Primates’ vertebrae are categorized into three types: proximal, transition, and distal^8,18–20^ (Fig. 1). The proximal vertebrae possess well-developed transverse processes, cranial and caudal zygapophyses and a neural arch. The transition vertebra also possesses transverse processes but has zygapophyses only cranially. The distal vertebrae have no zygapophyses, and lack neural arches. The three-division system recognizes the proximal region which covers the proximal vertebrae to transition vertebra (TV), the transitional region which includes the post-transition vertebra(e) caudally to the longest vertebra (hereafter, LV) and the distal region^16,18,19^ (Fig. 1). The vertebral length data that we collated from publications did not include information on the position of the transition vertebra. Therefore, we used a two-division system; which are the *proximal-transitional* (from the first caudal vertebra to the LV) and *distal* regions (Fig. 1).

The terminal vertebra was easily identifiable as it has an either rounded or pointed end on one side unlike non-terminal vertebrae, which have flat articular surfaces on both sides. Some small distal vertebrae could be lost during skeletal preparation. When we noticed missing vertebra(e), we estimated the number of missing vertebra(e) and their corresponding lengths using a linear regression model between the ordinal number and the vertebral length in the distal region (Supplementary Fig. S1). Vertebral lengths in the distal region decreased linearly and the length of the terminal vertebra was 1–2 mm^1,3,19^. Thus, we calculated a linear regression formula to every individual based on the distal caudal vertebrae. The numbers and lengths of the missing caudal vertebrae were estimated in 98 of the 141 individuals sampled. Accordingly, our samples were limited to those having two or more distal caudal vertebrae, and we did not use specimens that lacked an intermediate caudal vertebra(e). Estimated vertebral lengths of <1 mm were not included either to the length or to the numbers.

Ideally, each vertebral length should be standardized by HBL of the individual which it belongs to. However, only a small fraction of the specimens were associated with their own HBL records. Thus, we employed published HBL data for each taxon and sex^1–6^. We are reporting caudal vertebral lengths in percentages (see Table 2).

The sequential changes in caudal vertebral length can be described by a caudal vertebral length profile (CVL profile), whereby vertebral lengths (y-axis) are plotted against the ordinal number of caudal vertebrae (x-axis) (Supplementary Fig. S1a). CVL profile generally exhibits a lengthening from the first vertebra to the transitional region, where the LV appears as the vertex, and then a gradual shortening toward the distal end^1,3,4,18,34^, that is, representing an upward convex pattern. The CVL profiles are useful for comprehending lengths and ordinal numbers of caudal vertebrae simultaneously. Hereafter, an individual caudal vertebra will be referred to as Ca*n*, whereby *n* represents the ordinal number of the vertebra.

We checked the normality of distribution and the sex difference for the number of vertebrae in each region, total number of vertebrae, and lengths of the vertebrae in each taxon using the Shapiro-Wilk test, F-test, and two-sample t-test. Only *M*. *assamensis* showed statistically significant differences (number of caudal vertebrae in distal region: *t* = −3.553, df = 4.306, *P*-value = 0.021; total number of caudal vertebrae *t* = −3.164, df = 4.148, *P*-value = 0.03237) between the sexes for the number of caudal vertebrae in the distal region. Since previous studies have shown that RTL does not significantly differ between the sexes^1–6^, we pooled both sexes. Then we calculated mean length of each vertebra for each taxon (Supplementary Tables S1, S2). These values were used to draw vertebral profiles. The aspect ratio of CVL profile was calculated by LV length/median total number of caudal vertebrae using the value shown on Table 2. The higher aspect ratio means that the specie has higher CVL profile, which will lead to larger distal regional length. Lower aspect ratio species will have shorter distal regional length. The mean total lengths were calculated and then standardized by HBL (TotalL) shown in Supplementary Table S3. Then the mean single vertebral lengths were calculated by dividing the TotalL with the mean total number (TotalN) in Supplementary Table S3, and shown in Table 2.

Standard partial regression coefficients of multiple regression analysis were performed in order to investigate predictors of total tail length by comparing caudal vertebrae number and mean single vertebral length. Multiple regression analysis was done among all taxa, and each species group. Mean data of total caudal vertebral length/HBL (TotalL), single caudal vertebral length/HBL (SingleCVL), total number of caudal vertebrae (TotalN) of each taxon were used, and normalized into standard scores: zTotalL, zSingleCVL, zTotalN, which means are 0 and variance are 1 (Supplementary Table S3). Linear models were composed with zTotalL as a response variable and combinations of, zSingleCVL, and zTotalN as explanatory variables. For the analysis of each species group, the linear model formula was zTotalL = zSingleCVL + zTotalN,and the standard partial regression coefficients were compared.

Multiple comparisons of means by Tukey-Kramer contrasts were done to compare the LV length differences. This was the only analysis done on the raw measurement data, and the result is shown in Supplementary Fig. S3. We divided the examined taxa into three classes based on their RTLs, using a modified version of Russo and Shapiro’s classification^9^ to introduce a “medium” category; long-tailed, RTL > 100%; medium-tailed, 30–85%; and short-tailed, <20%, which Russo and Shapiro^9^ defined long-tailed for RTL > 100%; short-tailed, RTL = 37–100%; and very short-tailed, RTL < 0–15%.

The statistical analyses were conducted by using R studio and R Commander, and RExcel with significance of P < 0.05.

## Results

**Figure 2:**
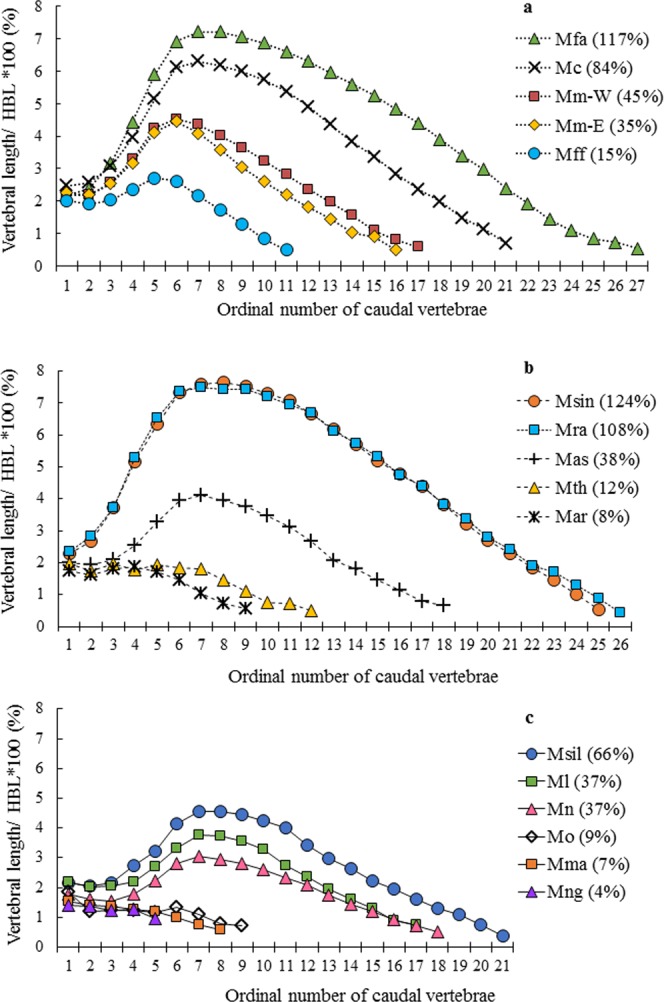
Mean caudal vertebral length profile in each *Macaca* species group. (**a**) *fascicularis* group; Mfa: *Macaca fascicularis*; Mc: *M*. *cyclopis*; Mm-W: western *M*. *mulatta*; Mm-E: eastern *M*. *mulatta*; and Mff: *M*. *f*. *fuscata*. (**b**) *sinica* group; Msin: *M*. *sinica*; Mra: *M*. *radiata*; Mas: *M*. *a*. *assamensis*; Mth: *M*. *thibetana*; and Mar: *M*. *arctoides*; (**c**) *silenus* group; Msil: *M*. *silenus*; Ml: *M*. *leonina*; Mn: *M*. *nemestrina*; Mo: *M*. *ochreata*; Mma: *M*. *maura*; and Mng: *M*. *nigra*. The relative tail lengths (RTLs) are shown in parentheses.

We compared the vertebral length and number by length profile (CVL profile) for each species group as shown in Fig. 2a–c. For the macaque species that have RTL of 15% or longer the vertebral length gradually increased from Ca1 to the LV, and then decreased gradually. The profiles exhibited an upward convex pattern (Fig. 2a–c). Shorter tail species (RTL ≤ 12%) have the profile of flat and decreased patterns, and LV was not always found in the intermediate position of the tail.

**Figure 3:**
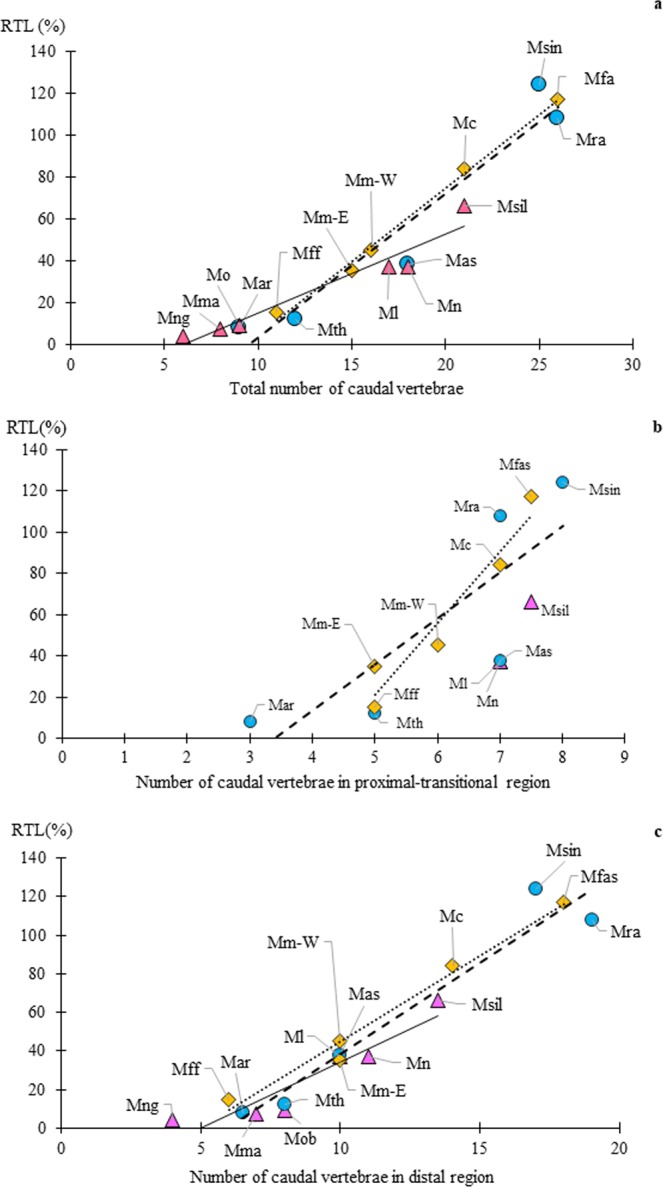
(**a**) Total number of caudal vertebra (x-axis) vs. relative tail length (RTL, y-axis) based on Table 2. Ordinary least squares regression lines were calculated in each species group and shown, which all species groups had good fitness (R^2^ ≥ 0.924). The formula and adjusted R^2^ are as follows; *fascicularis* group (dotted line): y = 7.027x − 65.88, R² = 0.994; *sinica* group (dashed line): y = 6.904x − 66.28, R² = 0.924; *silenus* group (line): y = 3.774x − 23.03, R² = 0.933. The *silenus* group has the smallest coefficient among the three species groups, and *fascicularis* and *sinica* groups were similar to each other. (**b**) Number of caudal vertebrae in proximal-transitional region (x-axis) vs. relative tail length (RTL, y-axis) based on Table 2. The lines are liner regression line for *fascicularis* group and *sinica* group. The formula and adjusted R^2^ are as follows; *fascicularis* group (dotted line): y = 34.596x − 151.84, R² = 0.9294; *sinica* group (dashed line): y = 22.375x − 76.25, R² = 0.6747. We did not calculate for *silenus* group because there are only two plots. (**c**) Number of caudal vertebrae in distal region (x-axis) vs. relative tail length (RTL, y-axis) based on Table 2. The lines are liner regression line for each species group and all species groups had good fitness (R^2^ ≥ 0.872). The formula and adjusted R^2^ are as follows; *fascicularis* group (dotted line): y = 8.875x − 43.75, R² = 0.9786; *sinica* group (dashed line): y = 9.4928x − 56.862, R² = 0.9427; *silenus* group: y = 6.8528x − 34.438, R² = 0.872. The *silenus* group has the smallest coefficient among the three species groups. Legends and abbreviations; ◇ (diamond): *fascicularis* group; ○ (circle): *sinica* group; △ (triangle): *silenus* group; Mff: *Macaca f*. *fuscata*; Mm-E: eastern *M*. *mulatta*; Mm-W: western *M*. *mulatta*; Mc: *M*. *cyclopis*; Mfa: *M*. *fascicularis*; Mar: *M*. *arctoides*; Mth: *M*. *thibetana*; Mas: *M*. *a*. *assamensis*; Mra: *M*. *radiata;* Msin: *M*. *sinica*; Mng: *M*. *nigra*; Mma: *M*. *maura*; Mo: *M*. *ochreata*; Ml: *M*. *leonina*; Mn: *M*. *nemestrina*; and Msil: *M*. *silenus*.

**Within** ***fascicularis*** **group**, all species had CVL profile of an upward convex pattern (Fig. 2a). The total number of vertebrae correlated with RTL, ranging from eleven (*M*. *fuscata*, RTL = 15%) to twenty-six (*M*. *fascicularis*, RTL = 117%) (Table 2, Fig. 3a). The eastern and western groups of *M*. *mulatta* showed quite resembling profiles in the proximal-transitional region, however, the western group showed a higher (up-shifted) profile in the distal region (Fig. 2a). The number of vertebrae in the proximal region was either four in shorter tail species (*M*. *fuscata* and *M*. *mulatta*) or five in longer-tail species (*M*. *fascicularis* and *M*. *cyclopis*) with inter-individual variation, three or six vertebrae respectively (Table 2). The number of vertebrae in the transitional region was commonly two (Table 2). Thus, the number of vertebrae in the proximal-transitional region (the position of LV) was greater with RTL increase, that is, five in *M*. *fuscata*, six in *M*. *mulatta*, seven in *M*. *cyclopis*, and seven or eight in *M*. *fascicularis* (Table 2, Fig. 3b). The number of distal vertebrae displayed much greater variation, ranging from six to eighteen, and correlated with RTL (Table 2, Fig. 3c).

**Figure 4:**
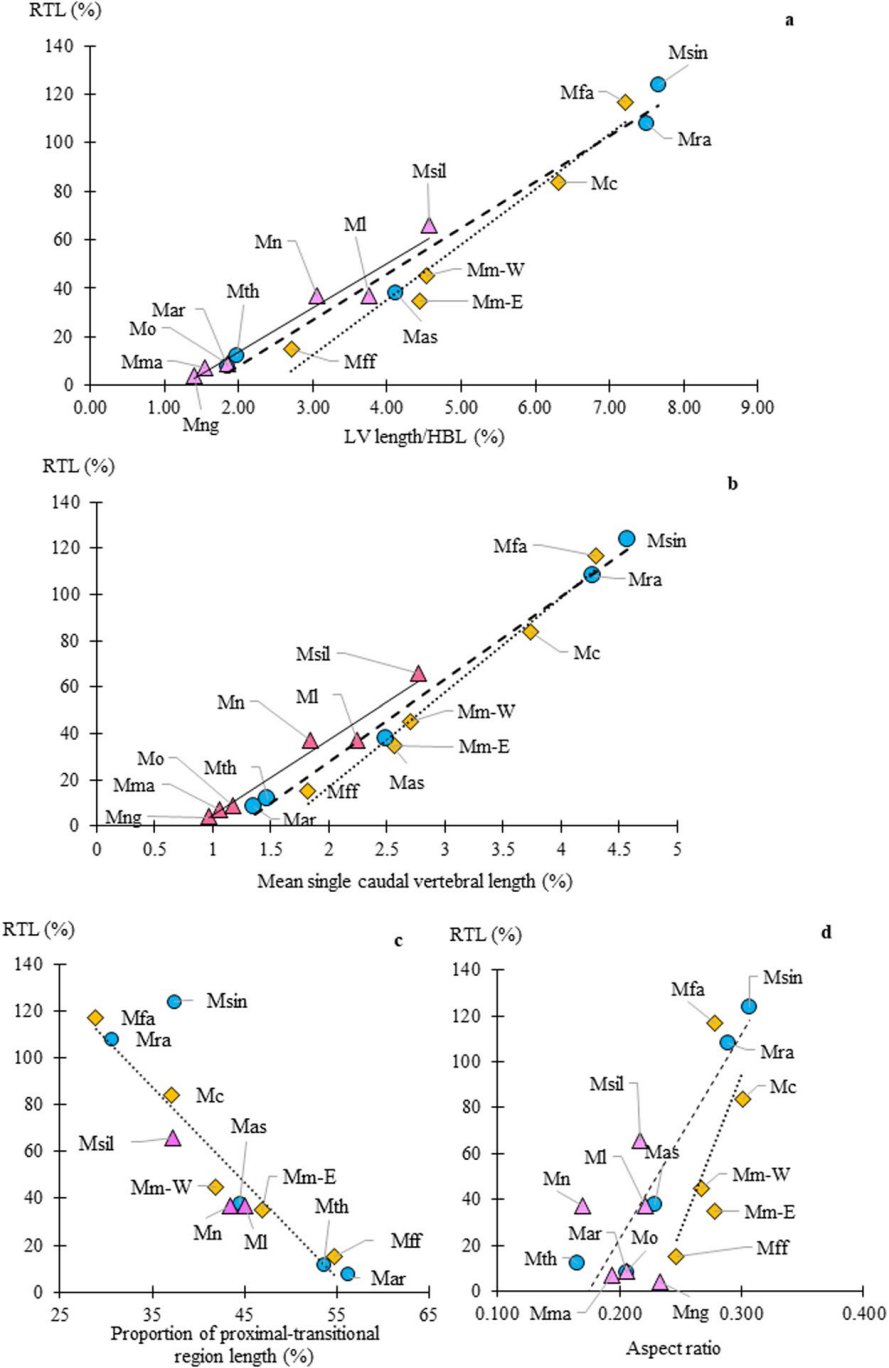
(**a**) LV length/ HBL (x-axis) vs. relative tail length (RTL, y-axis) based on Table 2. The lines are liner regression line for each species group and all species groups had good fitness (R^2^ ≥ 0.9548). The formula and adjusted R^2^ are as follows; *fascicularis* group (dotted line): y = 22.761x − 55.649, R² = 0.9575; *sinica* group (dashed line): y = 18.956x − 29.595, R² = 0.9814; *silenus* group (line): y = 18.321x − 22.745, R² = 0.9548. (**b**) Mean single caudal vertebra (x-axis) vs. relative tail length (RTL, y-axis) based on Table 2. Mean single vertebral length is the mean total length standardized by HBL, and divided by the median total number of caudal vertebrae. The lines are liner regression line for each species group. The formula and adjusted R^2^ are as follows; *fascicularis* group (dotted line): y = 41.07x −65.12, R^2^ = 0.984; *sinica* group (dashed line): y = 35.69x −43.33, R^2^ = 0.992; *silenus* group (line): y = 32.69x −28.24, R² = 0.964. (**c**) Proportion of the proximal-transitional region length (%, x-axis) vs. relative tail length (RTL, y-axis). The dotted line is liner regression formula of *fascicularis* group: y = −4.0782x + 230.02, R² = 0.9502. (**d**) Aspect ratio (x-axis) vs. relative tail length (RTL, y-axis). The lines are liner regression line and *sinica* group had higher R^2^ than *fascicularis* group. The formula and adjusted R^2^ are as follows; *fascicularis* group (dotted line): y = 1353.9x −311.71, R² = 0.4201; *sinica* group (dashed line): y = 890.38x −154.66, R² = 0.9143. The correlation was much less in *silenus* group (R² = 0.0029). Legends and abbreviations; ◇ (diamond): *fascicularis* group; ○ (circle): *sinica* group; △ (triangle): *silenus* group; Mff: *Macaca f*. *fuscata*; Mm-E: eastern *M*. *mulatta*; Mm-W: western *M*. *mulatta*; Mc: *M*. *cyclopis*; Mfa: *M*. *fascicularis*; Mar: *M*. *arctoides*; Mth: *M*. *thibetana*; Mas: *M*. *a*. *assamensis*; Mra: *M*. *radiata;* Msin: *M*. *sinica*; Mng: *M*. *nigra*; Mma: *M*. *maura*; Mo: *M*. *ochreata*; Ml: *M*. *leonina*; Mn: *M*. *nemestrina*; and Msil: *M*. *silenus*.

The length of LV correlated well with RTL, that is 2.71% (*M*. *fuscata*) to 7.22% (*M*. *fascicularis* (Table 2, Fig. 4a). Lengths of other vertebrae were proportionally similar. However, as a minor variation, Ca2 was slightly shorter than or equal to Ca1 in *M*. *fuscata* and *M*. *mulatta*, while in other species it increases from Ca1 (Fig. 2a). Mean single caudal vertebral length correlated well with RTL (Fig. 4b). Ordinary least squares regression lines were calculated in each species group, which all species groups had good fitness (adjusted coefficient of determination, R^2^ ≥ 0.964) (Fig. 4b). The proportion of the proximal-transitional region length ranged from 54.7% (*M*. *fuscata*) to 28.9% (*M*. *fascicularis*) of the total vertebral length, correlating well with RTL and represented by a regression line (y = −4.078 + 230.0, R^2^ = 0.950) (Fig. 4c). Western *M*. *mulatta* appeared to have a shorter proximal-transitional region than the estimation.

The aspect ratio of the CVL profile (LV length/total number of caudal vertebrae) also correlated with RTL for *fascicularis* and *sinica* groups (Table 2, Fig. 4d). However, *M*. *cyclopis* showed a higher ratio (0.301) than *M*. *fascicularis* (0.278), and its actual LV length had no significant difference with other long-tailed species (Supplementary Fig. S3). It is noteworthy that the *fascicularis* group tended to have high ratio, even *M*. *f*. *fuscata* had the ratio as high as 0.247 (Table 2).

**Within** ***sinica*** **group**, the long-tailed species (*M*. *radiata* and *M*. *sinica*) have similar characteristics to that of *M*. *fascicularis* in many parameters such as CVL profile (Fig. 2b, Table 2). The proximal vertebrae of *M*. *radiata* and *M*. *sinica* were relatively longer and their distal vertebrae were shorter than those of respective regions in *M*. *fascicularis* (Supplementary Fig. S2a). Thus, lengths of proximal-transitional regions were close to the regression of *fascicularis* group in *M*. *radiata* (Fig. 4c). The *M*. *sinica* deviated greatly from the regression probably because of individual variation as n = 1.

The LV of medium-tailed *M*. *assamensis* was at seventh like *M*. *radiata* despite its apparently shorter tail (Table 2). The length of LV (4.11%) was intermediate between those of *M*. *leonina*, *M*. *nemestrina* and *M*. *mulatta* of comparable RTLs (Supplementary Fig. S2c). The aspect ratio of CVL profile (Table 2, Fig. 4d) and proportion of proximal-transitional region length to RTL (Fig. 4c) were also intermediate between them.

The short-tailed *M*. *thibetana* (RTL = 12%) and *M*. *arctoides* (RTL = 8%) had CVL profiles of flat and decreased patterns, though there were inflection points in the profile, which appeared to demarcate the proximal-transitional from distal regions (Fig. 2b, Supplementary Fig. S2d). This may correspond to the caudal vertebral morphology; however it needs to be checked by specimens which are currently not available. The CVL profile pattern differed from those of Sulawesi macaques that do not show inflection points (Supplementary Fig. S2d).

**In** ***silenus*** **group** (Fig. 2c), the CVL profiles of medium-tailed species (*M*. *leonina*, *M*. *nemestrina*, and *M*. *silenus*) exhibit an upward convex pattern. The height of the profiles were lower than those of *fascicularis* group, especially in *M*. *nemestrina* as shown by the aspect ratio of 0.170 (Fig. 4a, Table 2, and Supplementary Fig. S2b,c). Although *M*. *leonina* have the same RTL with *M*. *nemestrina*, its profile was higher (aspect ratio = 0.222) than *M*. *nemestrina* (Table 2). The LV was positioned nearly constant around seventh in all medium-tailed species (Table 2), which was comparable to that of longer tail species of *fascicularis* group (*M*. *fascicularis* and *M*. *cyclopis*). Another characteristic of the *silenus* group was the short vertebrae in Ca2 and Ca3, even in *M*. *silenus* (RTL = 66%) had Ca2 and Ca3 shorter than its Ca1 (Fig. 2c).

Among the comparable total number of vertebrae species, the *silenus* group shows shorter RTL than those of *fascicularis* group, e.g., *M*. *silenus* vs *M*. *cyclopis*, *M*. *nemestrina* and *M*. *leonina* vs *M*. *mulatta* (Fig. 3a). This means that *silenus* group have a greater number of caudal vertebrae compared to *fascicularis* group to attain a given RTL. The ratios of proximal-transitional region length in these species were shorter than regression of *fascicularis* group, especially *M*. *nemestrina* (Fig. 4c).

The Sulawesi macaques (*M*. *nigra*, *M*. *maura*, *M*. *ochreata*) had characteristically short tails (RTL < 10%). The Ca1 was the longest and the CVL profiles of these macaques were flat and with decreased patterns. The LV which should be positioned distal to the proximal vertebrae at the transitional region had reduced its length.

**Standardized partial multiple regression analysis** was applied to see whether the length or the number of caudal vertebrae predict the total tail length. The linear models are listed in Tables 3 and 4. First, Models “1, 2 and 3” were done using all 16 taxa (Table 3) and had high adjusted R^2^ (≥0.8975). The standardized partial regression of single caudal vertebral length (zSingleCVL) was statistically significant, and total number of caudal vertebrae (zTotalN) was statistically significant when it was used alone (Model 3). Between the two parameters, the SingleCVL had the greater influence on the total tail length than TotalN (Table 3, Model 1 to 3). The Akaike’s Information Criterion (AIC) was smallest when zSingleCVL was used alone (Table 3, Model 2).

**Table 3:** Standard partial regression coefficient of multiple regression analysis.

Model	Standard partial regression coefficient	Remarks of t value and Pr of the coefficients			Adjusted R^2^	AIC
		zSingleCVL	zTotalN	Intercept		
1	zTotalL = 0.8429*zSingleCVL + 0.1525 *zTotalN + 1.75e-16	t > |2|, p***	t = 1.167, p = 0.264	t = 0, p = 1	0.9737	−8.141
2	zTotalL = 0.9873*zSingleCVL + 2.21e-16	t > |2|, p***	NA	t = 0, p = 1	0.973	−8.547
3	zTotalL = 0.951*zTotalN − 7.238e-17	NA	t > |2|, p***	t = 0, p = 1	0.8975	12.82

Key to significance level: t for t-value, *P < 0.05; **P < 0.01; ***P < 0.001.

**Table 4:** Each species group’s standard partial regression coefficient of multiple regression analysis.

Species Group	Standard partial regression coefficient	Remarks of t value and Pr of the coefficients			Adjusted R^2^
		zSingleCVL	zTotalN	Intercept	
SilG	zTotalL = 0.8432*zSingleCVL + 0.1602*zTotalN + 3.06e-16	t > |2|, p**	t = 1.186, p = 0.32094	t = 0, p = 1	0.9926
SinG	zTotalL = 0.9574*zSingleCVL + 0.03957*zTotalN + 2.24e-17	t > |2|, p = 0.125	t = 0.106, p = 0.925	t = 0, p = 1	0.9861
FasG	zTotalL = 0.2769*zSingleCVL + 0.7172*zTotalN + 2.37e-16	t = 0.301, p = 0.792	t = 0.779, p = 0.518	t = 0, p = 1	0.9733

Key to significance level: t for t-value, *P < 0.05; **P < 0.01; ***P < 0.001.

Next, each species group’s standard partial regression coefficient of multiple regression analysis was tested (Table 4) and had very high adjusted R^2^ (≥0.9733). In the case of the *silenus* group, the coefficient of zSingleCVL was 5.26 times greater than that of zTotalN, and the coefficient of zSingleCVL was statistically significant (Table 4, SilG). Similarly, the coefficient of zSingleCVL in the *sinica* group was 24.2 times greater than that of zTotalN, and the *t* value of zSingleCVL was statistically significant (Table 4, SinG). Conversely, in the *fascicularis* group, the coefficient of zTotalN was 2.59 times greater than zSingleCVL, though the *t* value and P-value were not statistically significant in either of the explanatory variables (Table 4, FasG). These results support that there are species group trends.

To summarize the results, in the *silenus* and *sinica* groups the length of caudal vertebrae was the major skeletal determinant, and tail length variation occurred by the changes in length more greatly than that in the numbers of vertebrae. Conversely, in the *fascicularis* group, the number of caudal vertebrae were the skeletal determinant, however, the two parameters are not mutually exclusive and both are working. The *fascicularis* group tends to have a high aspect ratio of CVL profile and longer mean single caudal vertebral length. On the other hand, the *silenus* group tended to have a low aspect ratio of CVL profile and shorter proximal caudal vertebrae. The *sinica* group showed the intermediate between the *fascicularis* and *silenus* groups.

## Discussion

The tail is constructed by the caudal vertebrae linearly arranged, and articulated by zygapophyseal and vertebral body joints. The tail extends and flexes by the accumulation of rotations and due to the range of rotation is limited in one joint. Proximal vertebrae have transverse processes and a spinous process, on which tail muscles insert, and consist of the main part of tail movement (swing). For these reasons previous studies focused on the number or length of the proximal caudal vertebrae and/or the morphology of the sacrum to find parameters that help estimate (reconstruct) the tail length^16,20,33^. However, our results showed the number and length of caudal vertebrae in the distal region varied more than the proximal region, and correlate more strongly with RTL. The proximal caudal vertebrae are limited in number, 4 or 5, and they are relatively short. Since the vertebral number relates with joints that makes the tail movement, there is less variation in numbers along with tail length. On the other hand, the distal region is long to obtain the magnitude of the moment of inertia. Vertebrae of transitional and distal regions are potentially necessary elements of cantilever where the proximal vertebra proportionally supports the mass of the consecutive adjacent distal vertebra. Due to this reason the longest (which is the largest) caudal vertebra (LV) is located at proximal 1/4 to 1/2 of tail, and the rest of vertebral length linearly decreases distally. The CVL profile can also describe the characteristics of the species’ tail function. The proximal-transitional region reflects the tail movement and flexibility^8,18,34^, and the distal region could reflects the balancing function. In species of RTL ≥ 15%, the CVL profile takes an upward convex pattern, which is a triangle-like shape, and the height (LV length) and length of the base (number of vertebrae) of the profile is larger in longer tail species. In short-tailed species with RTL ≤ 12%, the profile takes a flat and decrease pattern, which shows a tendency in smaller distal vertebrae. Meaning short-tailed species’ tails are able to flex-extend, but it hardly functions as a balancer.

We propose the aspect ratio of CVL profiles and tail length may provide a possible functional explanation of tail length adaptation in our target species. Among shorter medium-tailed species, the tails of *M*. *mulatta*, *M*. *a*. *assamensis*, and *M*. *leonina* are possible to function as mechanical assistance for arboreal positional behaviour, although the performance is not as great as that of long-tailed species. On the other hand, *M*. *nemestrina* have comparable RTL to that of *M*. *leonina* but its CVL profile is lower and its aspect ratio of the CVL profile is very low (Table 2, Supplementary Fig. S2c). We infer its tail does not function with balancing mechanism and this kind of aspect ratio could be one of the characteristics of terrestrial species. The aspect ratio of the CVL profile in *M*. *fuscata* is higher than that of *M*. *leonina*, however, the tail is too short to function as mechanical assistance for arboreality.

The trends of caudal vertebral number and length varied between species groups. Based on molecular phylogenetic studies, in the early stage of the Asian macaques’ evolution, the *silenus* group initially diverged from the *fascicularis*/*sinica* cluster, and then, the *fascicularis* and *sinica* groups diverged^10,26–28,35–38^. From this point, phylogeny diverged within the species group.

In *silenus* group, Ziegler *et al*.^39^ hypothesized that the ancestral population that was distributed in Sundaland became extinct, and then, some populations recolonized Sundaland from refugia of the Siberut Island. The proto-*nemestrina* diverged in Sundaland including Borneo, and then dispersed out of Sundaland into Indochina, and then into the Indian Peninsula to speciate *M*. *leonina* and *M*. *silenus*^39^. From the divergence of the *silenus* group, *M*. *nemestrina* could represent the state of common ancestor of the species group. The trend of *silenus* group is that the tail length is determined by the caudal vertebral length, low aspect ratio of CVL profile, and the Ca2 and Ca3 are shorter than Ca1. The numbers of caudal vertebrae are larger in the *silenus* group than that of species with comparable RTL of the *fascicularis* group. The flat and decrease pattern of CVL profile in Sulawesi macaques is the result of advancement of this trend, and these macaques are highly terrestrial^11^. On the other hand, arboreal species have increased the vertebral length during their evolution. *M*. *leonina* has the same RTL with *M*. *nemestrina* but inclines to arboreal^14,40^ and the CVL profile (Fig. 2c) is higher with one vertebra less. Arboreal adaptation is more advanced in *M*. *silenus*, and it has a longer tail with a higher profile than *M*. *leonina*. Tails were commonly reported to reduce in evolution^10,41^, however, Sehner *et al*. reported both increase and decrease occurred in macaques^29^. It is noteworthy that although the *silenus* group is distributed in a tropical area there are no extant long-tailed species, probably because they diverged from a medium tail length ancestral species. In addition, the short proximal caudal vertebrae (Ca2 and Ca3) could be effective to gain flexibility in the proximal region, which are the characteristics of this species group.

The long-tailed species (RTL > 100%): *M*. *fascicularis*, *M*. *radiata*, and *M*. *sinica* showed similar characteristics in our results, such as CVL profiles (Supplementary Fig. S2a). These three species diverged earlier than other species within each species group, they are currently distributed in lower latitudinal areas, and are inclined towards arboreality. Thus one possibility is that the common ancestor of these macaques could be long-tailed with similar CVL profiles to their current selves. Other species of these species groups have more or less reduced tails along with distribution to higher latitudinal areas and/or with inclination to terrestriality. Another possibility is that the common ancestor of these macaques was medium-tailed, and during the increase of relative tail length the caudal vertebral morphology converged. In this case, convergent evolution occurred probably related with the tail function for balancing mechanism.

In the *fascicularis* group, the *M*. *fascicularis* and *mulatta* subgroup diverged first^26–28,35,36,38,42^. The *M*. *mulatta* was classified into eastern and western groups by their tail length. The *M*. *cyclopis* and *M*. *fuscata* are closely related to eastern *M*. *mulatta*. From the view point of geographical distribution, proto-eastern *M*. *mulatta* distributed in the mainland of East Asia should be the common ancestor of this cluster. Thus, the proto-eastern *M*. *mulatta* and western *M*. *mulatta* first diverged within *mulatta* subgroup. Fooden and Albrecht^7^ suggested that the proto-eastern *M*. *mulatta* were widely distributed in the main land of East Asia, and had a cline in tail length; the southern population with long tails dispersed into Taiwan, and the northern ones with shorter tails dispersed into the Japanese Archipelago; and later, cline variation of tail length diminished in eastern *M*. *mulatta* by the retreat of northern populations back to the south during the glacial phase. The result of standardized partial regression analysis revealed that in the *fascicularis* group the number of the caudal vertebrae was mainly involved in the tail length variation. As the divergence occurs, the reduction in vertebral number occurred. This is why even short-tailed *M*. *fuscata* still shows the upward convex pattern in CVL profile, which is different from other short-tailed species. The aspect ratio of *M*. *cyclopis* is peculiar, which is higher than that of *M*. *fascicularis* which may suggest that vertebrae length extension occurred more than the increase of vertebral number in *M*. *cyclopis* during the evolution from *proto*-eastern *M*. *mulatta*, which is also similar to the evolution of *M*. *leonina* from proto-*nemestrina*.

The s*inica* group consists of long-tailed species ranging in South Asia, and medium- and short-tailed species in East Asia, Southeast Asia, and foothills of the Himalayan Range. *M*. *a*. *assamensis* and *M*. *thibetana* recently diverged through geographical isolation. The *M*. *arctoides* speciate from hybridization between the *mulatta* subgroup and the common ancestor of *assamensis*/*thibetana*^26^. The tail length reduction was more prone in vertebral length than in number in this species group.

Fooden suggested that the tail length may be controlled by homologous genetic factors among macaques^1^. However, our study showed that skeletal determinants of tail length were different among species groups. Sehner *et al*. reported the three separate clades of increase in relative tail length; (a) *M*. *nemestrina*, *M*. *silenus*, *M*. *pagensis*, and *M*. *leonine*; (b) *M*. *sinica*, *M*. *radiata*, and *M*. *munzala*; (c) *M*. *cyclopis*, *M*. *mulatta*, and *M*. *fascicularis*^29^, which exactly follows the species group; (a) *silenus* group, (b) *sinica* group, and (c) *fascicularis* group, and it supports our results. The number and length of vertebrae are determined in different developmental stages. In case of reducing the number of caudal vertebrae, it requires a mutation preventing the development of the somite, the base of the vertebrae, either during embryo development or at the stage of gene expression. In recent studies of developmental biology, it is reported that *Hox* genes relate to the regulation of the anterior-posterior axis of trunk morphogenesis^43^. Young *et al*.^44^ found that Hox and Cdx proteins control tail length extension and termination, and the number of somites relate to the number of caudal vertebrae. Conversely, changing caudal vertebral length occurs during the process of endochondral ossification that creates the vertebral skeleton and chondral bone^45,46^. First, during embryo development caudal vertebral length elongation occurs at the primary ossification center and at the growth plate. Second, after birth, each caudal vertebra forms a secondary ossification center, the diaphysis grows through epiphyseal cartilage proliferation, and growth stops through the loss of the epiphyseal growth plate and epiphysiodesis. The chondrocytes of epiphyseal cartilage are composed of reserve chondrocytes, proliferating chondrocytes, prehypertrophic chondrocytes, hypertrophic chondrocytes and endochondral bone; they divide, enlarge, die and are replaced by osteocytes^46^. The factors that together determine bone length, in this case the caudal vertebral length, are: the speed of division and length of time taken for division, the density of the cell and matrix, and the number of stem cells of chondrocytes and osteocytes. The caudal vertebral length differences among species and species groups could be elucidated by dissecting developmental stages of bone morphogenesis. Given that studies in mice have shown differentiation and growth to be controlled by: Indian hedgehog (Ihh), parathyroid hormone-related peptide (RTHrP), fibroblast growth factor (FGF), Sry-related HMG box 9 (Sox9), and runt-related gene2 (Runx2)^46–49^, we might expect these also to be the underling regulators in nonhuman primates.

In this way, the number of caudal vertebrae and the length of each caudal vertebrae are regulated by the actions of particular genes and developmental processes, therefore tail length evolution may be attributed to any changes in gene expression and function which lead to an alteration in development and growth. In the study of deer mouse, the genetic loci that control the number and length of caudal vertebrae were different to each other^24^. Thus, it is not entirely unexpected that there are different mechanisms for tail length evolution among species groups of macaques. Our study suggests that species group trends in the number and lengths of caudal vertebrae are retained in each species group. Future research is needed to reveal what exactly determines species group uniqueness, focusing on molecular and developmental mechanisms.

## Supplementary information

Supplementary Information
