# Supplementary material for: Skeletal determinants of tail length are different between macaque species groups
Source: Sci Rep. 2019 Feb 4;9:1289. doi: 10.1038/s41598-018-37963-z (PMC6362266; doi:10.1038/s41598-018-37963-z)
Supplement: Supplementary file 1 — Supplementary Information [file 41598_2018_37963_MOESM1_ESM.pdf]

Skeletal determinants of tail length are different between macaque species groups

Hikaru Wakamori*[1], Yuzuru Hamada[1]

Author affiliation: 1. Primate Research Institute, Department of Biology, Faculty of Science, Kyoto University, Inuyama, Aichi, 484-8506, Japan

Address: Primate Research Institute, Kyoto University, Inuyama, Aichi, 484-8506, Japan

Telephone number: +81-568-63-0567, Fax number: +81-568-63-0085

E-mail address: wakamori.hikaru.26a@kyoto-u.jp

Three supplementary tables and three supplementary figures are provided in the following pages.

Supplementary Table S1: Mean length of caudal vertebrae (mm) for each region, and mean relative tail length (RTL).

Species group	*Macaca* spp.	Regions				Total[*8]	RTL (%)
		proximal	transitional	proximal-transitional[*7]	distal		
fascicularis	*M. fuscata* [#1]	45.5	14.8	60.3	49.9	110.2	15[*1]
	M. mulatta (East)	50.4	42.5	92.9	105.2	198.1	35[*2]
	(West)	52.4	44.9	97.4	135.2	232.6	45[*2]
	M. cyclopis	87.0	62.8	149.8	254.1	403.9	84[*1]
	M. fascicularis	79.2	62.1	141.3	348.2	489.5	117[*1]
sinica	*M. arctoides*			35.5	27.7	63.2	8[*3]
	M. thibetana			53.7	46.6	100.3	12[*4]
	M. assamensis [#2]	66.2	45.0	111.2	138.8	250.0	38[*4]
	M. radiata			179.4	406.7	586.1	108[*4]
	M. sinica			200.3	335.5	535.8	124[*4]
silenus	*M. nigra*			7.3	25.0	32.3	4[*5]
	M. maura			9.3	45.4	54.7	7[*5]
	M. ochreata [#3]			10.9	51.7	62.6	9[*5]
	M. leonina			85.8	104.5	190.3	37[*6]
	M. nemestrina	51.0	33.3	84.4	110.0	194.4	37[*6]
	M. silenus			111.8	188.9	300.7	66[*6]

The subspecies are as follows; #1: *M. f. fuscata*; #2: *M. a. assamensis*; #3: *M. ochreata brunnescens*.
The references and mean RTL were cited and calculated from the literature as follows.

*1: Fooden, 2006; *2: Fooden, 2000; *3: Fooden, 1990; *4: Fooden, 1988; *5: Fooden, 1969; *6: Fooden 1975.

*7: Proximal-transitional region is calculated by summing up each mean vertebral length until the median number of proximal-transitional region which is shown in Table 2.

*8: Total is calculated by summing up each mean vertebral length until the median total number which is shown in Table 2.

Supplementary Table S2. Mean standardized length of caudal vertebrae for each region, and mean relative tail length (RTL).

Species group	*Macaca* spp.	Regions				Total*8	RTL (%)
		proximal	transitional	proximal-transitional*7	distal		
fascicularis	*M. fuscata* #1	0.0832	0.0271	0.1104	0.0913	0.2017	15*1
	M. mulatta (East)	0.1016	0.0857	0.1872	0.2119	0.3992	35*2
	(West)	0.1025	0.0879	0.1904	0.2644	0.4548	45*2
	M. cyclopis	0.1720	0.1243	0.2963	0.5026	0.7989	84*1
	M. fascicularis	0.1805	0.1414	0.3219	0.7934	1.1153	117*1
sinica	*M. arctoides*			0.0704	0.0550	0.1254	8*3
	M. thibetana			0.0940	0.0816	0.1755	12*4
	M. assamensis #2	0.1186	0.0805	0.1990	0.2486	0.4476	38*4
	M. radiata			0.3560	0.8069	1.1628	108*4
	M. sinica			0.4280	0.7168	1.1448	124*4
silenus	*M. nigra*			0.0140	0.0482	0.0622	4*5
	M. maura			0.0155	0.0757	0.0913	7*5
	M. ochreata #3			0.0185	0.0876	0.1061	9*5
	M. leonina			0.1822	0.2218	0.4039	37*6
	M. nemestrina	0.0895	0.0585	0.1480	0.1929	0.3410	37*6
	M. silenus			0.2103	0.3553	0.5656	66*6

The subspecies are as follows; #1: *M. f. fuscata*; #2: *M. a. assamensis*; #3: *M. ochreata brunnescens*.
The references and mean RTL were cited and calculated from the literature as follows.

*1: Fooden, 2006; *2: Fooden, 2000; *3: Fooden, 1990; *4: Fooden, 1988; *5: Fooden, 1969; *6: Fooden 1975.

*7: Proximal-transitional region is calculated by summing up each mean vertebral length until the median number of proximal-transitional region which is shown in Table 2.

*8: Total is calculated by summing up each mean vertebral length until the median total number which is shown in Table 2.

Supplementary Table S3: Values used for standard partial regression coefficient of multiple regression analysis.

Species group	*Macaca* spp.	TotalL[*1]	Total N[*2]	SingleCVL[*3]	SpG	Normalize into standard scores		
						zTotalL	zTotalN	zSingleCVL
fascicularis	*M. fuscata* [#1]	0.2034	11.16	0.0182	Fas	-0.7000	-0.7799	-0.5305
	M. mulatta (East)	0.3980	15.54	0.0256	Fas	-0.1867	-0.1048	0.0808
	(West)	0.4521	16.70	0.0271	Fas	-0.0440	0.0737	0.2024
	M. cyclopis	0.7997	21.37	0.0374	Fas	0.8729	0.7925	1.0610
	M. fascicularis	1.1080	25.75	0.0430	Fas	1.6861	1.4677	1.5252
sinica	*M. arctoides*	0.1216	8.88	0.0136	Sin	-0.9159	-1.1317	-0.9107
	M. thibetana	0.1646	11.25	0.0147	Sin	-0.8025	-0.7658	-0.8201
	M. assamensis [#2]	0.4191	16.84	0.0250	Sin	-0.1311	0.0951	0.0314
	M. radiata	1.1121	26.00	0.0428	Sin	1.6968	1.5062	1.5040
	M. sinica	1.1448	25.00	0.0458	Sin	1.7831	1.3522	1.7543
silenus	*M. nigra*	0.0610	6.25	0.0097	Sil	-1.0756	-1.5360	-1.2374
	M. maura	0.0920	8.75	0.0106	Sil	-0.9938	-1.1509	-1.1593
	M. ochreata [#3]	0.1061	9.00	0.0118	Sil	-0.9567	-1.1124	-1.0648
	M. leonina	0.4010	17.92	0.0225	Sil	-0.1788	0.2611	-0.1802
	M. nemestrina	0.3339	18.15	0.0184	Sil	-0.3558	0.2970	-0.5168
	M. silenus	0.5833	21.00	0.0278	Sil	0.3020	0.7360	0.2606
	Average	0.4688	16.22	0.0246	Average	0.0000	0.0000	0.0000
	Standard deviation (SD)	0.3791	6.49	0.0121	SD	1.0000	1.0000	1.0000

The subspecies are as follows; #1: *M. f. fuscata*; #2: *M. a. assamensis*; #3: *M. ochreata brunnescens*.

*1: The mean total caudal vertebral length/HBL (TotalL) is the average of total length standardized by head and body length.

*2: Total N is species' mean total number of caudal vertebrae.

*3: The mean single caudal vertebral length/HBL (SingleCVL) is mean total length standardized by HBL divided by species' mean total number.

Supplementary Figure S1

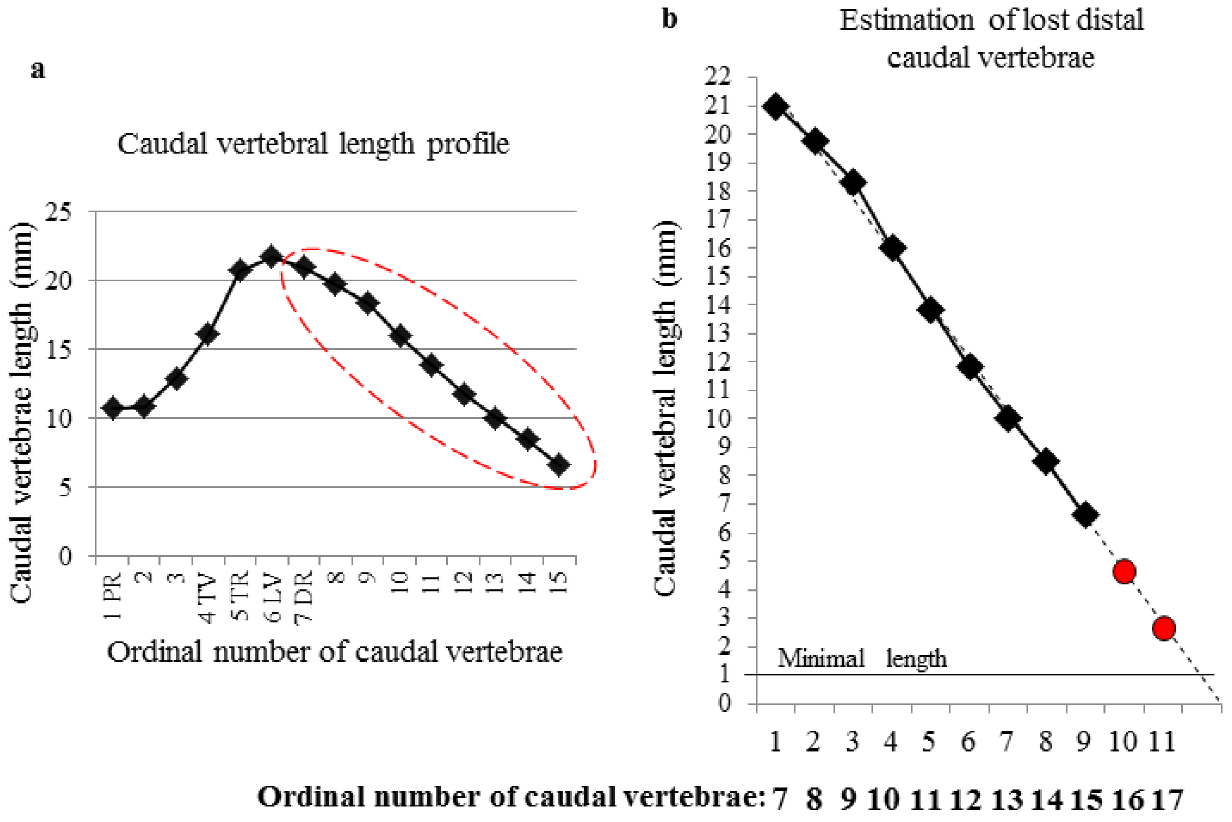

Figure legend

Supplementary Figure S1. (a)Example of caudal vertebral length profile (CVL profile), and (b) estimation of lost distal caudal vertebrae. This is a case of male western *M. mulatta*, with 15 caudal vertebrae preserved as skeletal specimens. In this case the Ca4 was the transition vertebra (TV) and the Ca6 was the LV, thus proximal region is from first to Ca4, transitional region is from Ca5 to Ca6, and distal region is from Ca7 to the terminal vertebra. The most distal vertebra was Ca15, which length was 6.63 mm and it was not the terminal vertebra. Therefore we estimate the lost distal vertebrae. We extract the distal caudal vertebral length, surrounded by a dotted red ellipse in the Supplementary Fig. S1a, and provide scatter plot and liner regression line (dotted line in Supplementary Fig. S1b). The regression line formula was: $y = -1.8683x + 23.341$ with satisfied R^2 value ($R^2 = 0.9961$). This formula was used to calculate the lost caudal vertebrae's length and number by substituting $x = 10, 11, 12, \ldots$ As a result, the calculated vertebral lengths were 4.66 mm in Ca16, 2.79 mm in Ca17, and 0.921 mm in Ca18. The length of Ca18 was shorter than 1 mm thus it is not included in the estimation. Therefore, we added two estimated caudal vertebrae, Ca16 and Ca17, to this individual, which is shown with red circles in Supplementary Fig. S1b.

Supplementary Figure S2

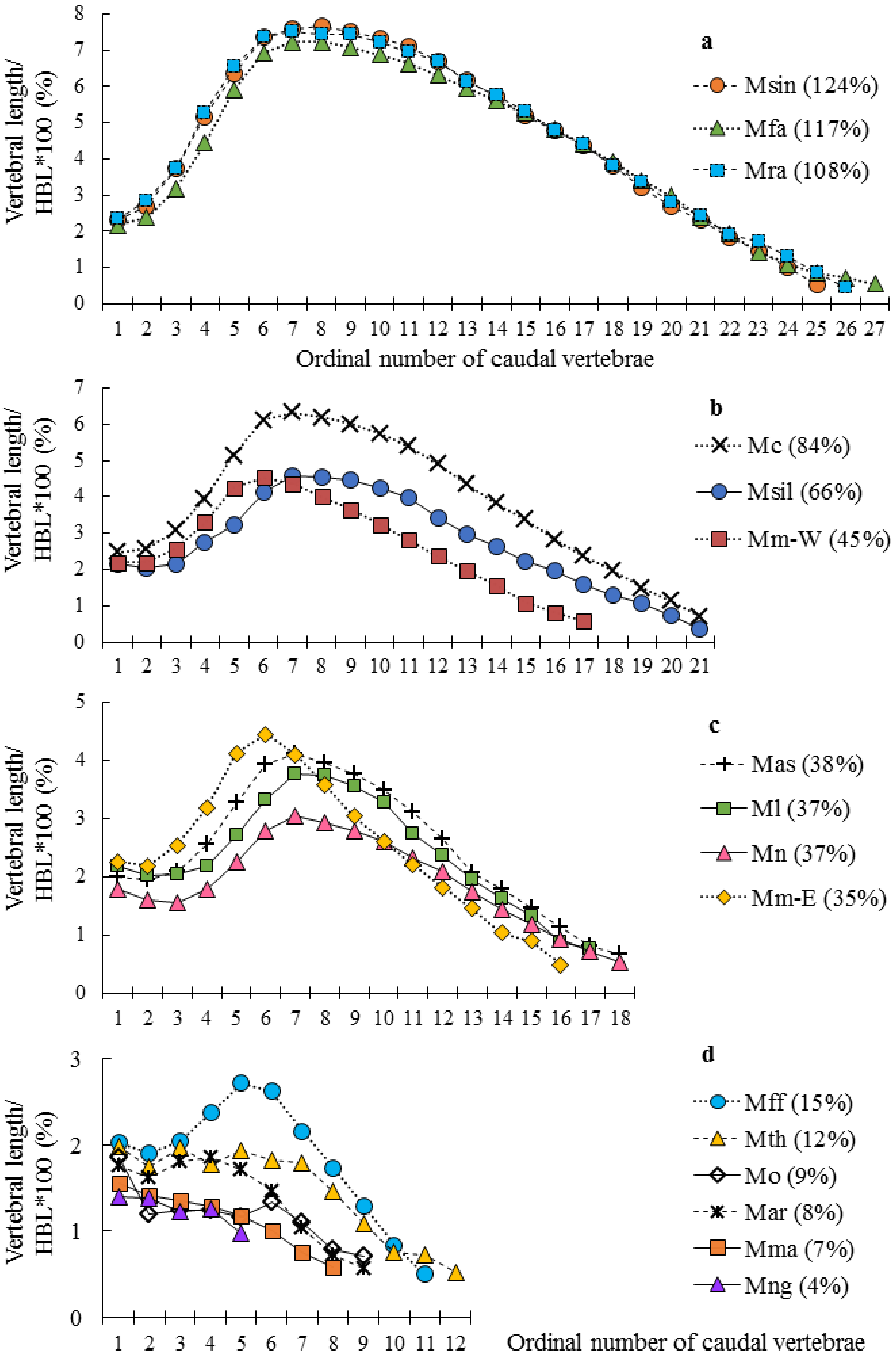

Figure legend

Supplementary Figure S2. CVL profile shown by similar relative tail length (RTL) classes. (a) Long-tailed species (RTL > 100%), Msin: *Macaca. sinica*; Mfa: *M. fascicularis*; Mra: *M. radiata*, (b): longer medium-tailed species (RTL 45-85%), Mc: *M. cyclopis*; Msil: *M. silenus*; Mm-W: western *M. mulatta*, (c): shorter medium-tailed species (RTL 35-38%), Mas: *M. a. assamensis*; Ml: *M. leonina*; Mn: *M. nemestrina*; Mm-E: eastern *M. mulatta*; (d): short-tailed species (RTL 4-15%), Mff: *M. f. fuscata*; Mth: *M. thibetana*; Mo: *M. ochreata*; Mar: *M. arctoides*; Mma: *M. maura*; Mng: *M. nigra*. The relative tail lengths (RTLs) are shown in parentheses.

Supplementary Figure S3

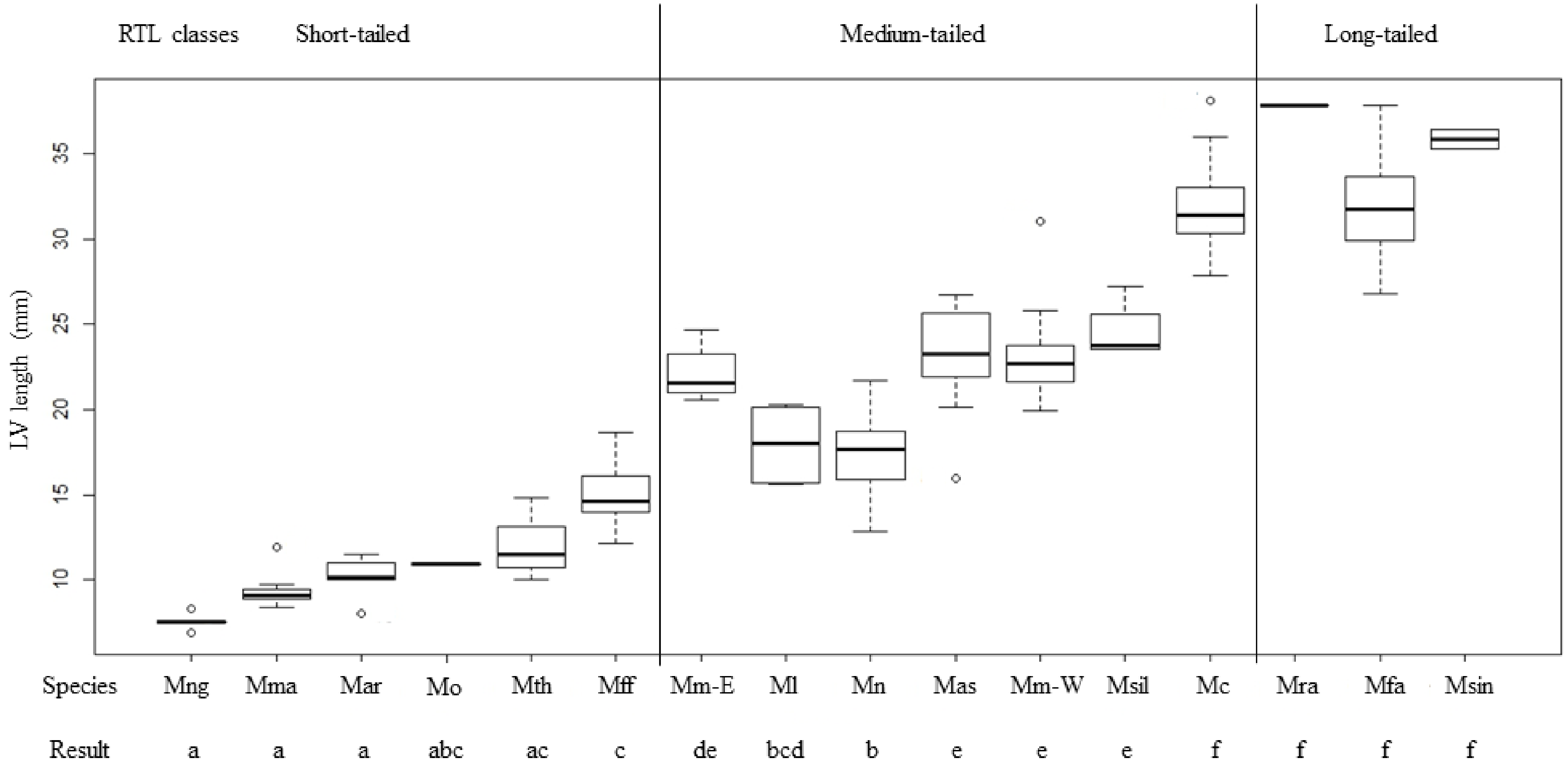

Figure legend

Supplementary Figure S3. Boxplot of LV length on raw data, and result of multiple comparisons of means by Tukey-Kramer contrasts. The alphabets in the line “Result” shows the significant difference among species. The same alphabet is given when there is no significant difference between means. The ascending order of the alphabet corresponds to the average ascending order. Mng: *M. nigra*; Mma: *M. maura*; Mar: *M. arctoides*; Mo: *M. ochreata*; Mth: *M. thibetana*; Mff: *Macaca fuscata fuscata*; Mm-E: eastern *M. mulatta*; Ml: *M. leonina*; Mn: *M. nemestrina*; Mas: *M. a. assamensis*; Mm-W: western *M. mulatta*; Msil: *M. silenus*; Mc: *M. cyclopis*; Mra: *M. radiata;* Mfa: *M. fascicularis*, and Msin: *M. sinica*.
